# DNA methylation patterns associated with breast cancer prognosis that are specific to tumor subtype and menopausal status

**DOI:** 10.3389/fgene.2023.1133443

**Published:** 2023-03-02

**Authors:** Do Hyun Kim, Alexandra M. Binder, Hua Zhou, Su Yon Jung

**Affiliations:** ^1^ Department of Biostatistics, Fielding School of Public Health, University of California, Los Angeles, Los Angeles, CA, United States; ^2^ Cancer Epidemiology Program, University of Hawaii Cancer Center, Honolulu, HI, United States; ^3^ Department of Epidemiology, Fielding School of Public Health, University of California, Los Angeles, Los Angeles, CA, United States; ^4^ Department of Computational Medicine, University of California, Los Angeles, Los Angeles, CA, United States; ^5^ Translational Sciences Section, School of Nursing, University of California, Los Angeles, Los Angeles, CA, United States; ^6^ Jonsson Comprehensive Cancer Center, University of California, Los Angeles, Los Angeles, CA, United States

**Keywords:** DNA methylation signatures, breast cancer, subtypes, menopause, survival and prognosis

## Abstract

Tumor subtype and menopausal status are strong predictors of breast cancer (BC) prognosis. We aimed to find and validate subtype- or menopausal-status-specific changes in tumor DNA methylation (DNAm) associated with all-cause mortality or BC progression. Associations between site-specific tumor DNAm and BC prognosis were estimated among The Cancer Genome Atlas participants (*n* = 692) with Illumina Infinium HumanMethylation450 BeadChip array data. All-cause mortality and BC progression were modeled using Cox proportional hazards models stratified by tumor subtypes, adjusting for age, race, stage, menopausal status, tumor purity, and cell type proportion. Effect measure modification by subtype and menopausal status were evaluated by incorporating a product term with DNAm. Site-specific inference was used to identify subtype- or menopausal-status-specific differentially methylated regions (DMRs) and functional pathways. The validation of the results was carried out on an independent dataset (GSE72308; *n* = 180). We identified a total of fifteen unique CpG probes that were significantly associated ( $\left.P\leq 1\times 10^{-7}\right)$ with survival outcomes in subtype- or menopausal-status-specific manner. Seven probes were associated with overall survival (OS) or progression-free interval (PFI) for women with luminal A subtype, and four probes were associated with PFI for women with luminal B subtype. Five probes were associated with PFI for post-menopausal women. A majority of significant probes showed a lower risk of OS or BC progression with higher DNAm. We identified subtype- or menopausal-status-specific DMRs and functional pathways of which top associated pathways differed across subtypes or menopausal status. None of significant probes from site-specific analyses met genome-wide significant level in validation analyses while directions and magnitudes of coefficients showed consistent pattern. We have identified subtype- or menopausal-status-specific DNAm biomarkers, DMRs and functional pathways associated with all-cause mortality or BC progression, albeit with limited validation. Future studies with larger independent cohort of non-post-menopausal women with non-luminal A subtypes are warranted for identifying subtype- and menopausal-status-specific DNAm biomarkers for BC prognosis.

## Introduction

Breast cancer (BC) is the most frequently diagnosed cancer in women. In 2020, BC accounted for 6.9% (approximately 2.3 million cases) of new cancer deaths globally (Sung et al., 2021). Advancements in screening and treatment options have contributed to improvements in BC prognosis (Goldhirsch et al., 2011). Epigenetic dysregulation is a hallmark of cancer (Jones et al., 2016) and thus epigenetic patterns may serve as predictors of risk and prognosis to guide more personalized treatment plans to further improve patient outcomes. Both *in vitro* and population-based studies have implicated aberrant histone and DNA methylation (DNAm) in BC tumorigenesis and progression (Dumont et al., 2008; Elsheikh et al., 2009; Stone et al., 2015). Several population-based epigenomic studies of BC tissues have investigated the prognostic utility of DNAm at specific CpG loci as the potential candidate biomarker for BC survival (Hao et al., 2017; Xiao et al., 2018; de Almeida et al., 2019; Du et al., 2019; Liu et al., 2020; Tao et al., 2020). These studies have demonstrated a selected panel of CpG loci can potentially be used to distinguish high- and low-risk groups for all-cause mortality among BC patients, improving prediction of BC prognosis.

However, prior studies of the DNAm and BC survival have focused on identifying a panel of prognostic CpG probes that are agnostic to BC molecular subtypes or menopausal status (Hao et al., 2017; de Almeida et al., 2019; Du et al., 2019; Liu et al., 2020; Tao et al., 2020). BC is heterogeneous cancer with different intrinsic subtypes defined by female hormone-receptor status (Koboldt et al., 2012). Subtypes are distinguished using gene expression arrays or immunohistochemistry, and include luminal A, luminal B, HER-2, normal-like and basal-like. BC prognosis differs by the subtypes (Byler et al., 2014; Ohnstad et al., 2017; Yang and Polley, 2019), which have guided the use of subtype-specific therapeutic regimens (Goldhirsch et al., 2011). Indeed, Xiao et al. examined DNAm sites associated with all-cause mortality among subjects with luminal BC; however, they did not examine DNAm of subjects with basal-like subtype or based on menopausal status (Xiao et al., 2018). Pre-menopausal women have a higher proportion of tumor subtypes associated with poorer prognosis (e.g., basal-like and HER2), compared to post-menopausal women (Keegan et al., 2012). Moreover, the prognosis for pre-menopausal women with luminal A or luminal B subtype is worse than that of post-menopausal women with the same subtype (Lian et al., 2017). Thus, prognoses of patients with BC could differ by their menopausal status in conjunction with their molecular subtypes. DNAm may provide a means to capture these differences in biology that are linked to BC outcomes.

In this study, we aimed to identify DNAm sites associated with BC survival outcomes in breast tumor tissue, which are specific to different BC subtypes or menopause status. We evaluated these associations among The Cancer Genome Atlas (TCGA) BC participants with Illumina Infinium HumanMethylation450 BeadChip (450K) array data obtained from breast tumor tissue samples. This subset of the TCGA BC participants had a median overall follow-up time of 27.7 months, which is considered relatively a short follow-up time for survival analysis (Liu et al., 2018). Thus, in addition to analyzing overall survival (OS), we analyzed progression-free interval (PFI), which was defined as the period between the date of diagnosis and the date of first new tumor event. We conducted an epigenome-wide association study (EWAS) of these outcomes, accounting for potential effect measure modification by BC subtypes or menopausal status. We further validated these associations in an independent cohort of 180 subjects with breast tumor tissue samples. To the best of our knowledge, this is the first study that has investigated subtype- or menopausal-status-specific DNAm sites that are associated with all-cause mortality and BC progression. Identified DNAm sites will potentially enable clinicians to provide more precise prognosis for BC patients.

## Materials and methods

### Study population

The breast cancer data set of 862 samples and corresponding Infinium 450K DNAm data were obtained from TCGA Genomic Data Commons (GDC), which houses molecular profiles of different cancer types from the TCGA project. The data set included four different survival outcomes, including overall survival, progression-free interval, disease-specific survival, and disease-free interval. These outcomes were defined and curated using available clinical information by Liu et el. and were made available in GDC (Liu et al., 2018). BC subtypes of samples were classified based on Spearman distance between samples’ expression profiles and 50-gene centroids (representing different subtypes) constructed using the Prediction Analysis of Microarray (PAM) algorithm (Tibshirani et al., 2002). Inclusion criterion was availability of OS or PFI endpoints. Exclusion criteria were based on tumor types, stages and missingness of covariates which were included in the model. Specifically, we excluded subjects whose menopausal status (*n* = 80), age (*n* = 1), race (*n* = 16) and tumor stage (*n* = 7) information were missing. We also excluded subjects with stage four cancer (*n* = 16), and adjacent normal samples (*n* = 110) and HER2 subtype (*n* = 46). We randomly chose one sample from a subject if the subject had multiple samples. Subjects whose race was not white were categorized as other, and whose stage was not three was categorized as stage one or two. The menopausal status was categorized into two categories, post-menopause, or pre- or peri-menopause. For this study, we analyzed overall survival and progression-free interval outcomes.

In the end, our study included 692 breast tumor samples from female TCGA BC participants. A majority of these participants were post-menopausal (Table 1). Tumor subtypes of TCGA participants were previously classified using the 50-gene PAM50 model (Parker et al., 2009; Koboldt et al., 2012), with 415 samples classified as luminal A, 141 as luminal-B, and 136 as basal-like (Table 1). During follow-up, 87 participants died, and 94 progressed to a new tumor event. The median follow-up time was 42 months (IQR = 55) for OS endpoint and 27 months (IQR = 36) for PFI endpoint (Table 1).

**TABLE 1:** Summary of subject characteristics.

Variable	TCGA ( $\left.n=692\right)$	GEO $\left(n=180\right)$	*p*-value^4^
Age			0.148
< 60	373	108	
≥ 60	318	72	
Missing	1	0	
Race			
White	515	-	
Other	163	-	
Missing	14	-	
Stage			
I & II	500	-	
III	174	-	
Missing	18	-	
Menopause status			
post-menopause	449	-	
Pre & peri-menopause	174	-	
Missing	69	-	
Subtype			< 0.001
luminal A	415	52	
luminal B	141	63	
Basal	136	65	
Purity^2^	0.643 (0.170)	0.595 (0.125)	< 0.001
Cell Proportion^2^			
CE1	0.114 (0.213)	0.058 (0.147)	< 0.001
CE2	0.025 (0.087)	0.044 (0.098)	0.013
CE3	0.106 (0.166)	0.125 (0.178)	0.281
CE4	0.063 (0.123)	0.051 (0.109)	0.142
CE5	0.166 (0.210)	0.106 (0.200)	< 0.001
NE	0.06 (0.132)	0.069 (0.116)	0.704
St	0.116 (0.142)	0.168 (0.168)	< 0.001
Im	0.109 (0.170)	0.143 (0.266)	0.001
OS			
Event^3^	87 (13%)	29 (16%)	0.213
Follow-up time^1^	42 (55)	42 (37)	0.789
PFI			
Event^3^	94 (12%)	-	
Follow-up time^1^	27 (36)	-	

CE, cancer epithelia cell; NE, normal epithelial cell; St, stromal cell; Im, immune cell; OS, overall survival; PFI, progression-free interval;

^1^ Median follow-up time in months (IQR);

^2^ Median (IQR);

^3^ Count (Percentage);

^4^ Pearson's Chi-squared test or Wilcoxon rank sum test

### Preparation of DNAm data

We used minfi (Version 1.34.0) in R (Aryee et al., 2014) to normalize, correct for background noise, and convert the raw signal intensities into beta-values. The raw DNAm data was preprocessed using Noob (normal-exponential out-of-band) background correction with dye-bias normalization in minfi. We included 485,512 CpG loci in our analysis of the TCGA and GEO datasets.

### Estimation of cell purity and cell proportions

The purity of each tumor sample was estimated using the InfiniumPurify (Version 1.3.1) R package (Qin et al., 2018). First, the top 1000 informative, differentially methylated CpG sites (iDMCs) were selected using the rank-sum test to detect significant differences in methylation levels (beta-values) between tumor and normal samples. Additionally, the variances of methylation levels of tumor samples were required to be greater than 0.005 for iDMCs. Next, iDMCs were categorized as hypermethylated if the mean beta-values of the tumor samples were greater than the normal samples, and into the hypomethylated group, otherwise. Beta-values of hypomethylated iDMCs were transformed into 1− beta-values, and those of hypermethylated iDMCs were identity transformed. The distribution of the transformed beta-values was estimated using the Gaussian kernel, of which the mode was taken to be estimated tumor purity. The tumor type was set as breast cancer because no normal samples were used.

The cell-type proportions of each sample was estimated using the RefFreeEWAS (Version 2.2) R package (Houseman et al., 2014). The cell proportions for each sample were estimated by non-negative matrix factorization (NMF), which decomposes the beta matrix into a cell-type-specific methylation matrix and a cell proportion matrix. We used an existing cell-type-specific methylation matrix to initialize the NMF rather than estimating it *de novo*. This cell-type-specific methylation matrix was previously estimated using the informative probes selected based on reference DNA methylation profiles of known cell lines, and the TCGA breast cancer data set (Onuchic et al., 2016). Eight cell types were inherited from the initial cell-type specific methylation matrix, and thus no estimation of the number of cell types was conducted. The number of iterations was set to 100. The final cell-type-specific methylation matrix did not differ much from the initial cell-type-specific methylation matrix.

### Site-specific analysis

All statistical analyses were performed using R. The site-specific analysis was conducted using TCGA data. PH assumptions were assessed by examining log-log survival curves of OS and PFI for covariates including age, ethnicity, stage, menopausal status, and subtype. The log-log survival curves of PFI and OS for subtype overlapped noticeably across different subtypes. For this reason, Cox PH models was stratified by tumor subtype, allowing the baseline hazards functions to vary across the subtypes. The association between DNAm and our outcomes were assessed for each probe separately, with beta-values standardized across samples, such that the effect sizes corresponded to a one standard deviation increase in DNAm. All models were adjusted for age, race, stage, menopause status, tumor purity, and cell type proportion. For each model, the genome-wide significance level of $1\times 10^{-7}$ was established using Bonferroni correction to account for multiple testing of 485,512 probes. Model A tested the association of beta-values and the survival outcome stratified by the tumor subtypes. Model B assumed the association between beta-values and the survival outcome could depend on tumor subtype by integrating a product term between subtype and site-specific DNAm. Thus, under this model, we were able to compute the estimates of log hazards and their P -value for each of the subtype. Model C assumed the association between beta-values and the survival outcome could depend on menopausal status (post-menopausal vs. pre- and peri-menopause), by including a product term between menopausal status and DNAm. As in Model B, we were able to compute the estimates of log hazards and their P -values for each menopausal status. During the model fitting, a small number of probes produced warnings due to a lack of convergence. These probes were excluded from further analysis.

### Functional pathway and differentially methylated region analysis

We used the functional class scoring (FCS) robust rank aggregation approach implemented in the methylGSA (Version 1.6.1) R package (Ren and Kuan, 2019) for the pathway analysis. This approach uses the site-specific EWAS inference to identify gene sets enriched among probes that are associated with survival outcomes. Two types of biases should be corrected for functional pathway analysis with DNAm data. One is the length bias arising from differing lengths of pre-defined gene sets based on molecular and cellular functions, which is addressed by FCS *via* Gene Set Enrichment Analysis (Subramanian et al., 2005). The other is the probe bias arising from different numbers of probes mapped to gene sets of equal size, which is addressed by the robust rank aggregation (RRA). RRA computes combined *p*-value based on the order statistic of *p*-values of probes (from site-specific analyses) mapped to a gene, assuming independent identical uniform distribution of *p*-values. We restricted our evaluation of enrichment to gene sets containing between 100 and 500 genes. The adjusted *p*-values of gene sets (enriched pathways) account for multiple hypothesis testing and the size of gene sets. We defer readers to (Subramanian et al., 2005) for details on calculation of adjusted *p*-values.

The ipDMR approach implemented in the Enmix (Version 1.25.1) R package (Xu et al., 2020) was used to identify differentially methylated regions (DMRs). The initial DMRs are identified as a region bound by a pair of adjacent probes across the methylome. These regions are filtered based on their sizes and combined P -values, each of which is computed as some function of *p*-values of probes (from site-specific analyses) within a region. We defer readers to (Xu et al., 2020) for details on the form of this function that computes the combined *p*-values. The initial DMRs go through another round of filtering and merging regions based on their sizes, distances, and adjusted combined P -values. A DMR was deemed significant if its adjusted combined P -value was less than 0.05.

### Validation analysis

We used the Illumina 450K array data of 295 BC tumor tissue samples available in GEO database (GSE72308) to validate site-specific analysis results. The samples were obtained from retrospectively selected frozen samples and prospective cohorts who were treated with adjuvant and neoadjuvant therapies at Jules Bordet Institute from 1995 to 2009 (Jeschke et al., 2017). IHC-based tumor subtypes available in the dataset were taken as the subtypes of the samples. To be consistent with site-specific analysis of TCGA data, we removed samples of HER2 subtype. OS endpoint was the only available outcome that was in common with survival outcomes available in TCGA data. Age and tumor subtype were the only available covariates in the data set. Tumor purity and cell type proportion were estimated using identical methods in the site-specific analysis. The beta-values of each probe were normalized across samples before conducting EWAS. Since menopausal status information was not available, Model C was not fitted. Model A and Model B were identical to the ones fitted using TCGA data except for the covariates such as race, stage, and menopausal status, which were not available in the GEO data set. EWAS results from each model were compared to the results from TCGA site-specific analyses. In particular, log hazards of the probes were compared to examine whether the directions of the association between beta-values to survival outcomes were similar between TCGA and GEO EWAS results.

## Results

### Site-specific results

**FIGURE 1:**
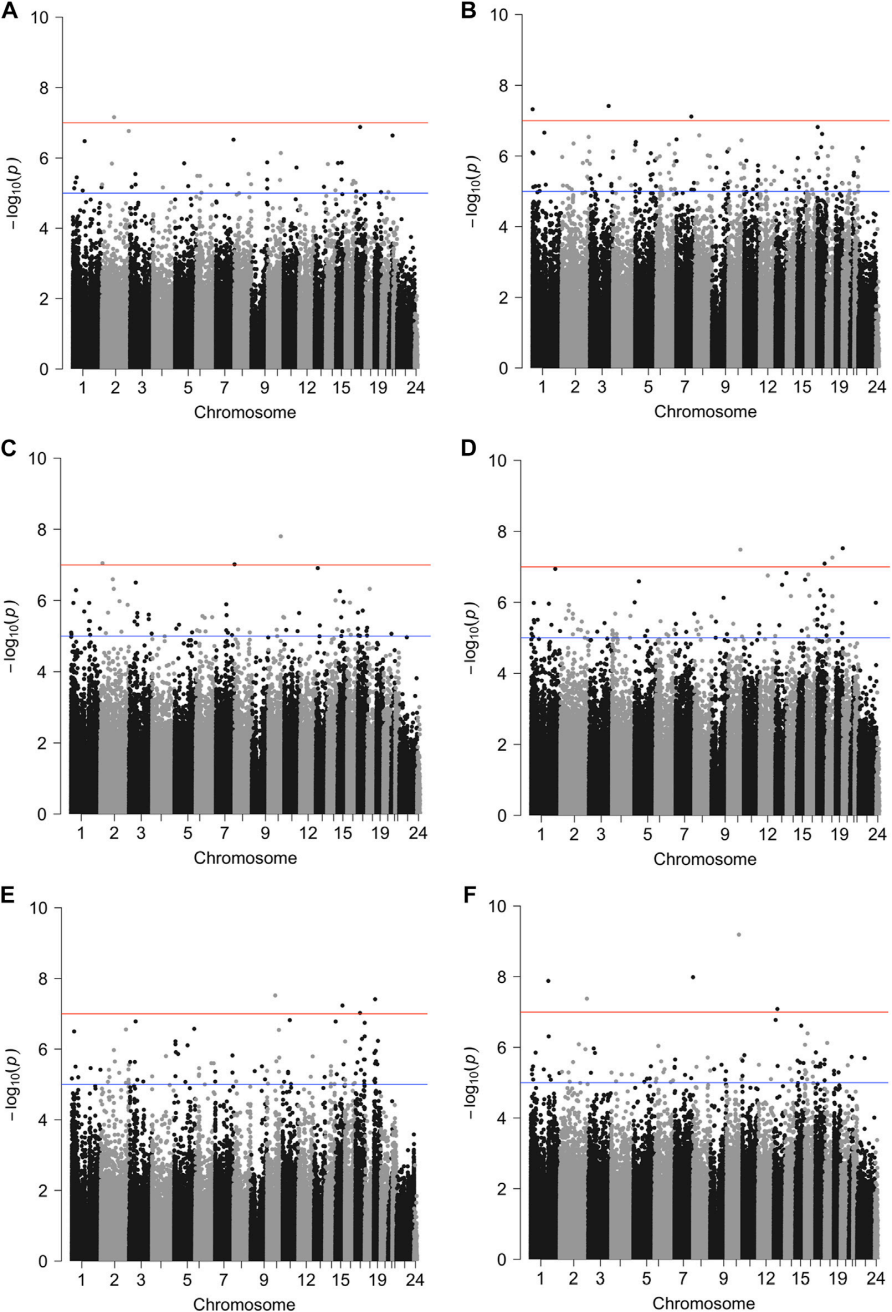
Manhattan plots of genome-wide associations between DNAm levels and survival outcomes. We employed Models A, B and C to investigate CpG probes associated with survival outcomes (overall survival and progression free interval) in a subtype or menopausal status specific manner. Model A tested the association of beta-values and the survival outcomes stratified by the tumor subtypes (no effect measure modifications); Model B tested the association between beta-values and the survival outcomes under the assumption that the association could be modified by tumor subtype; Model C tested the association between beta-values and the survival outcomes under the assumption that the association could depend on menopausal status (post-menopause vs. pre- and peri-menopause). All models adjusted for age, race, stage, menopausal status, tumor purity and cell type proportion. Here, we show Manhattan plots of genome-wide associations with at least one genome-wide significant CpG probe. The red line indicates a genome-wide significant level of $1\times 10^{-7}$ . The blue line indicates a *p*-value threshold of $1\times 10^{-5}$ . **(A)** Overall survival, Model A; **(B)** overall survival, Model B (luminal A); **(C)** progression-free interval, Model A; **(D)** progression-free interval, Model B (luminal A); **(E)** progression-free interval, Model B (lumina-B); **(F)** progression-free interval, Model C (post-menopause).

**FIGURE 2:**
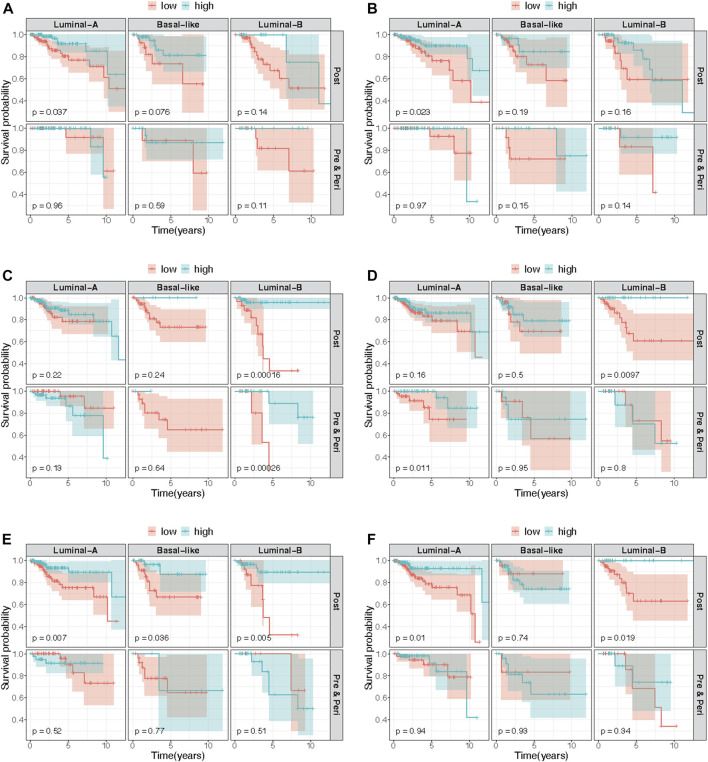
Kaplan-Meier curves comparing estimated survival probabilities between low and high methylation groups, stratified by both tumor subtype and menopausal status. We selected stratified Kaplan-Meir curves of top six probes based on *p*-values of stratified log-rank test (not shown) among the CpG probes that met genome-wide significance level of $1\times 10^{-7}$ in the site-specific analyses: **(A)** cg04921068 (luminal A specific) associated with overall survival; **(B)** cg15462203 (luminal A specific) associated with overall survival; **(C)** cg22776912 (luminal B specific) associated with progression free interval; **(D)** cg15348839 (post-menopause specific) associated with progression free interval; **(E)** cg12511487 (post-menopause specific) associated with progression free interval; **(F)** cg16976520 (post-menopause specific) associated with progression free interval. Each panel is a stratum created by stratifying by both tumor subtype (luminal A, luminal B and basal-like) and menopausal status (post-, and pre- and peri-menopausal status). The low and high methylation groups were categorized with respect to median beta-values of the probe. The 95% confidence interval of survival probability at each time point is represented by the shaded region.

The association between site-specific DNAm and each clinical outcome was assessed across methylome. Time to event was modeled using Cox proportional hazards (PH) models, stratified by tumor subtype, and adjusted for age, race, stage, menopausal status, DNAm-estimated tumor purity and cell proportions (8 cell types). Based on this model, one probe was associated with OS at genome-wide significance (*p* < $1\times 10^{-7}$ ; Figure 1; Table 2, Supplementary Figure S1). Effect measure modification by tumor subtype or menopausal status was evaluated by further incorporating product terms between these characteristics and DNAm. Three additional luminal A subtype-specific probes met genome-wide significance. These probes were significantly associated with OS only after accounting for effect measure modification by tumor subtype. For all four probes, an increase in DNAm was associated with a lower hazard of all-cause mortality. All four probes were located in open sea regions and none of the probes were located within promoter regions (Table 2). Kaplan-Meier (KM) plots for the probes presented in Table 2 are presented in Figure 2, stratified by both tumor subtype and menopause status, and with site-specific DNAm dichotomized by the median. These results were consistent with the direction of associations estimated from the site-specific Cox PH models.

**TABLE 2:** Genome-wide significant CpG probes associated with survival outcomes specifically to tumor subtype or menopausal status of women.

Overall Survival							
Probe	Chr	Position	*P* -value	HR (95% CI)	Island Context	Gene Region	Gene
**Model A**							
cg03985718	2	105924245	6.91E-08	0.48 (0.37-0.63)	OpenSea	Body	*TGFBRAP1*
**Model B (luminal A)**							
cg04921068	3	160787668	3.84E-08	0.12 (0.06-0.25)	OpenSea	3’ UTR	*PPM1L*
cg15462203	1	1277499	4.75E-08	0.52 (0.41-0.66)	OpenSea	Body	*DVL1*
cg17827670	7	129008130	7.65E-08	0.10 (0.04-0.23)	OpenSea	1^st^ Exon; Body; 5’ UTR	*AHCYL2*

**Progression Free Interval**							
**Probe**	**Chr**	**Position**	***P* -value**	**HR (95% CI)**	**Island Context**	**Gene Region**	**Gene**
**Model A**							
cg09926728	10	105519992	1.57E-08	0.59 (0.49-0.71)	OpenSea	Body	*SH3PXD2A*
cg18703983	2	18097337	8.97E-08	0.53 (0.42-0.67)	OpenSea	5’ UTR	*KCNS3*
cg16976520	7	158588852	9.62E-08	0.52 (0.41-0.66)	OpenSea	Body	*ESYT2*
**Model B (luminal A)**							
cg17735983	19	59074482	3.01E-08	2.44 (1.78-3.35)	Island	Body	*MZF1;LOC100131691*
cg09926728	10	105519992	3.28E-08	0.58 (0.48-0.70)	OpenSea	Body	*SH3PXD2A*
cg10678486	18	48494218	5.47E-08	1.84 (1.48-2.30)	N Shore	TSS200	*ELAC1*
cg13447284	17	63116336	8.11E-08	0.48 (0.37-0.63)	N Shelf		
**Model B (luminal B)**							
cg24328142	10	71234693	3.04E-08	0.33 (0.22-0.49)	OpenSea	Body	*TSPAN15*
cg03216043	19	10928639	3.87E-08	0.15 (0.08-0.29)	OpenSea	Body;TSS1500	*DNM2;MIR199A1*
cg22776912	15	81630789	5.85E-08	0.17 (0.09-0.32)	OpenSea	Body	*TMC3*
cg06956006	17	40066945	9.47E-08	0.14 (0.07-0.29)	OpenSea	Body	*ACLY*
**Model C (post-menopause)**							
cg09926728	10	105519992	6.44E-10	0.57 (0.48-0.68)	OpenSea	Body	*SH3PXD2A*
cg16976520	7	158588852	1.03E-08	0.46 (0.36-0.60)	OpenSea	Body	*ESYT2*
cg00175150	1	150486261	1.32E-08	0.49 (0.39-0.63)	N Shore	3’UTR	*ECM1*
cg15348839	2	232224846	4.19E-08	0.53 (0.43-0.67)	OpenSea		
cg12511487	13	50418481	8.23E-08	0.39 (0.28-0.55)	N Shelf		

We conducted genome-wide association analysis between DNAm levels and survival outcomes (overall survival and breast cancer progression), using TCGA breast cancer DNAm data. A stratified Cox PH (by tumor subtype) was used to model the association. Model A tested the association of beta-values and the survival outcomes stratified by the tumor subtypes (no effect measure modifications); Model B tested the association between beta-values and the survival outcomes under the assumption that the association could be modified by tumor subtype; Model C tested the association between beta-values and the survival outcomes under the assumption that the association could depend on menopausal status (post-menopause vs pre- and peri-menopause). All models adjusted for age, race, stage, menopausal status, tumor purity and cell type proportion. The genome-wide significance level was $1\times 10^{-7}$ . Chr, chromosome; HR, hazards ratio; CI, confidence interval; UTR, untranslated region; TSS200, 0-200 bp upstream of transcription start site; TSS1500, 200-1500 bp upstream of transcription start site.

Similar models were used to identify site-specific associations with PFI. A majority of genome-wide significant sites were found to be luminal A, luminal B and/or post-menopausal specific (Figure 1; Figure 2; Table 2, Supplementary Figure S1). There were 10 probes that were significantly associated with PFI only after accounting for effect measure modification by tumor subtype or menopausal status. Higher DNAm was associated with a reduced hazard of disease progression for all but two probes, cg17735983 and cg10678486, both of which were specific to the luminal A subtype (Table 2). While most of the probes were in open sea regions and gene bodies, cg17735983 was located in an CpG island, and cg10678486 was located within 200 bp of the transcription start site (TSS200) of *ELAC1*. Probe cg09926728 was associated with PFI overall, but this association was strongest among participants with the luminal A subtype and postmenopausal women (Supplementary Figure S5I). The estimated hazard ratios (HRs) for this probe were similar across different models ranging between 0.57–0.59. Similarly, higher methylation at cg16976520 was associated with a reduced hazard of progression overall, which was strongest among the post-menopausal women (Figure 2F; Supplementary Figure S5M). Among post-menopausal women, the rates of progression were higher among women with the luminal A and luminal B subtypes with lower methylation at cg16976520, relative to those with the basal-like subtype (Figure 2F).

### Functional pathway analysis results

We evaluated gene ontology (GO) enrichment among the site-specific associations with BC outcomes, taking into account the length bias of a gene and the number of probes per gene (Supplementary Figures S2 and Supplementary Figure S3). For each endpoint, GO enrichment was assessed based on the overall model, as well as models incorporating effect modification by either subtype or menopausal status. The number of significantly (adjusted *p*-value ≤0.05 ; Materials and methods section) enriched pathways ranged from 7 to 155 for OS, and from 0 to 217 for PFI. While the ten most significantly enriched GOs differed between each model, there were some overlap between significantly enriched GOs by different prognosis outcomes and different strata. The GOs related to axon development were associated with OS among both post-menopausal, and pre- and peri-menopausal women. These GOs were also associated with PFI for both luminal A subtypes and post-menopausal women. The GOs related to insulin regulation (GO:0050796 and GO:0030073) were significantly associated (adjusted *p*-value ≤0.05 ) with OS and suggestively associated (adjusted *p*-value ≈0.1 ) with PFI among pre- and peri-menopausal women. The GOs related to regulation of neuron death were associated with PFI for the luminal B subtype.

### Differentially methylated region analysis

**FIGURE 3:**
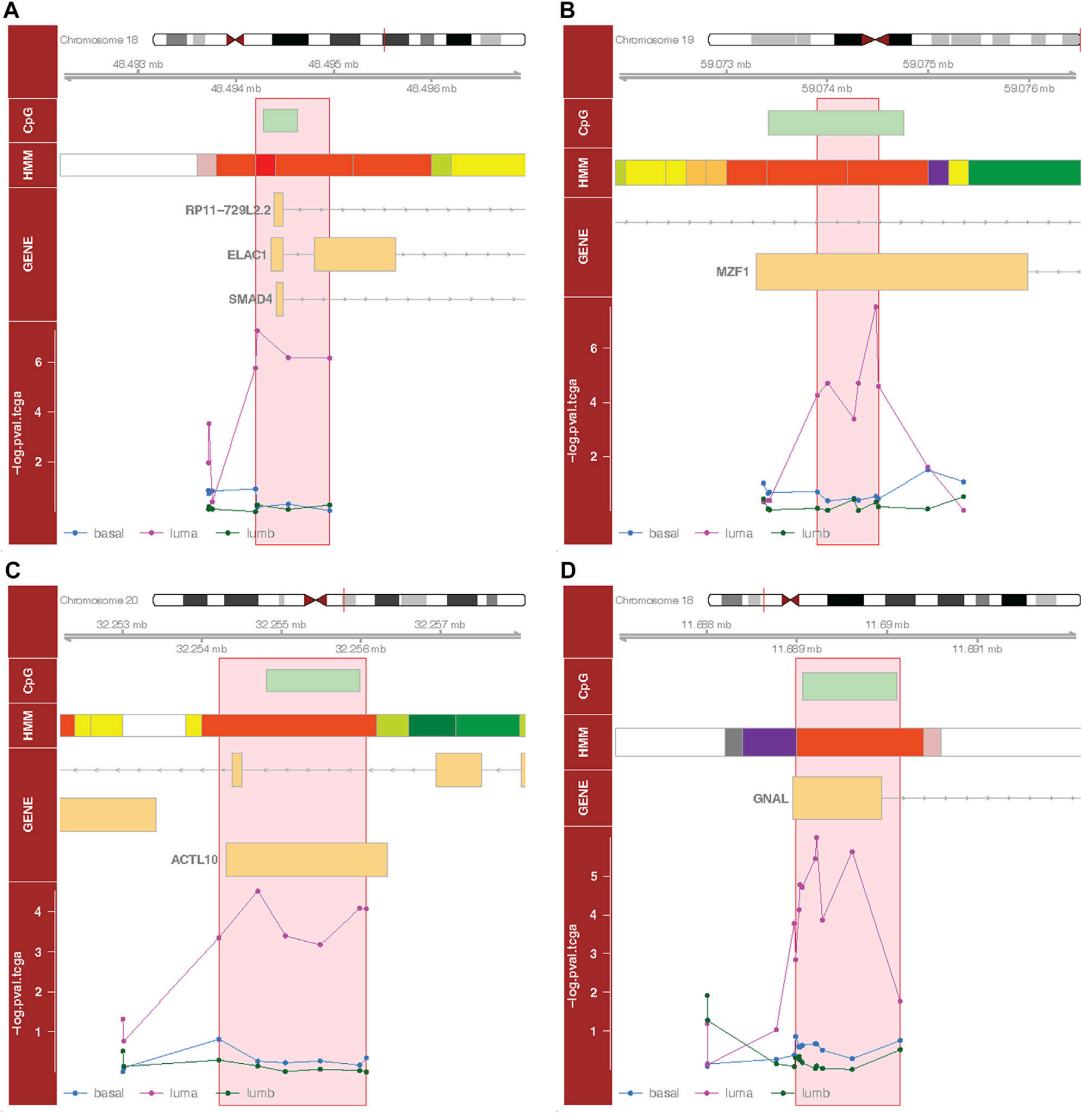
Selected differentially methylated regions (DMRs). Each subplot depicts a region in the genome around identified DMR (flanking 2000 bp). Here, we show the top four DMRs based on adjusted *p*-values (See Materials and Methods), containing at least four CpG probes. **(A)** Luminal A-specific DMR (chr18:48494200–48494958) associated with progression free interval; **(B)** luminal A-specific DMR (chr19:59073901–59074507) associated with progression free interval; **(C)** luminal A-specific DMR (chr20:32254215–32256071) associated with progression free interval; **(D)** luminal A-specific DMR (chr18:11688987–11690145) associated with overall survival. The first track shows the CpG island context; the second track shows the chromatin state; the third track shows the gene context (Ensembl); the fourth track shows the *p*-values of the probes (from the site-specific analyses) for each subtype. Red boxes represent DMRs. Vertical bars on the chromosome schematic locate plotted regions. Chromatin state color scheme: electric lime, transcribed 3′ preferential and enhancer (Enh) or transcribed 5′ preferential and Enh or transcribed and weak Enh; red, active transcription start site (TSS); orange red, promoter (Prom) upstream/downstream TSS; yellow, weak Enh or primary H3K27ac possible Enh; white, quiescent; orange, active Enh; pink, poised Prom; dark purple, bivalent Prom; light green, weak transcription; green, transcribed or strong transcription; gray, repressed polycomb.

To identify DMRs associated with our outcomes, we applied an approach that integrates site-specific inference between proximal loci. Significant DMRs differed by tumor subtype and menopausal status (Table3; Table 4; Figure 3, Supplementary Table S1, Supplementary Figure S4). These included a DMR within the promoter of *ELAC1* that was associated with PFI among the luminal A tumor samples, but not among the luminal B and basal-like samples (Figure 3A). In particular, this DMR was the most significant among all DMRs containing at least four probes, and included a genome-wide significant CpG probe, cg10678486, which was identified in the site-specific analysis to be associated with PFI for women with luminal A tumor subtype. We identified 271 luminal A specific DMRs that were associated with OS (Table 3). Only one out of three genome-wide significant luminal A subtype-specific probes associated with OS appeared within these significant DMRs. Similarly, only one of the three probes significantly associated with PFI was within one of the 42 DMRs associated with PFI (Table 3). These results indicated a majority of DMRs were not driven by one strong site-specific association, but several weaker associations in the same direction among proximal loci (Table 4). Several DMRs were not close to known genes. For example, an identified DMR specific to luminal A subtype did not reside near any known gene, but in a region indicated by chromatin state of promoter upstream TSS (Supplementary Table S1, Supplementary Figure S4F).

**TABLE 3:** Summary of genomic regions associated with breast cancer prognosis.

Event	Model^3^	# of Probes^1^	Size^1,2^	# of DMR	# of GWS Probes in DMR	# of GWS Probes
OS	Model A	2 (0.5)	123 (55)	3	0	1
OS	Model B (luminal A)	2 (6)	187 (824)	271	1	3
OS	Model B (luminal B)	2 (0)	217 (34)	2	0	0
OS	Model C (post-menopause)	3 (0)	109 (35.5)	6	0	0
PFI	Model A	2 (7.25)	201.5 (283.75)	42	1	3
PFI	Model B (luminal A)	2 (8)	186 (1505)	65	2	4
PFI	Model B (luminal B)	2 (6)	202 (418.5)	71	1	4
PFI	Model B (basal-like)	2 (0)	104 (110)	5	0	0
PFI	Model C (post-menopause)	2 (2)	132 (479.75)	72	2	5
PFI	Model C (pre- & peri-menopause)	2 (0)	160 (0)	1	0	0

Subtype- or menopausal-status-specific genomic regions associated with survival outcomes were identified using the summary statistics (*P*-values) results from the site-specific analysis of TCGA breast cancer DNAm data. OS, overall survival; PFI, progression-free interval; DMR, differentially methylated region; GWS, genome-wide significant ( $1\times 10^{-7})$ . ^1^Median (IQR); ^2^Size represents the number of base-pairs in a DMR; ^3^Models A, B and C correspond to models used in the site-specific analyses. Model A tested the association of beta-values and the survival outcomes stratified by the tumor subtypes (no effect measure modifications); Model B tested the association between beta-values and the survival outcomes under the assumption that the association could be modified by tumor subtype; Model C tested the association between beta-values and the survival outcomes under the assumption that the association could depend on menopausal status (post-menopause vs pre- and peri-menopause). All models adjusted for age, race, stage, menopausal status, tumor purity and cell type proportion.

**TABLE 4:** The top four differentially methylated regions containing at least four CpG loci.

PFI, Model B (luminal A), chr18:48494200 – 48494958^1^ (corresponding to Figure 3A)						
Probe	Position	*P*-value	HR(95% CI)	Island Context	Gene Region	Gene
cg16121470	48494201	1.72e-06	1.82 (1.42-2.32)	N Shore	TSS200	ELAC1
cg10678486	48494218	5.47e-08	1.84 (1.48-2.30)	N Shore	TSS200	ELAC1
cg22532061	48494536	6.67e-07	1.56 (1.31-1.86)	Island	5'UTR	ELAC1
cg21461196	48494958	6.80e-07	1.89 (1.47-2.42)	S Shore	5'UTR	ELAC1

PFI, Model B (luminal A), chr19:59073901 – 59074507^1^ **(corresponding to** Figure 3B **)**						
Probe	Position	*P*-value	HR(95% CI)	Island Context	Gene Region	Gene
cg26482665	59073902	5.55e-05	1.99 (1.42-2.78)	Island	Body	LOC100131691;MZF1
cg05014211	59074005	1.99e-05	2.12 (1.50-2.99)	Island	Body	LOC100131691;MZF1
cg19363466	59074265	4.15e-04	1.89 (1.33-2.69)	Island	Body	LOC100131691;MZF1
cg08447733	59074308	1.96e-05	2.15 (1.51-3.05)	Island	Body	LOC100131691;MZF1
cg17735983	59074482	3.01e-08	2.44 (1.78-3.35)	Island	Body	LOC100131691;MZF1
cg00577109	59074507	2.55e-05	1.99 (1.44-2.74)	Island	Body	LOC100131691;MZF1

PFI, Model B (luminal A), chr20:32254215 – 32256071^1^ **(corresponding to** Figure 3C **)**						
Probe	Position	*P*-value	HR(95% CI)	Island Context	Gene Region	Gene
cg12710480	32254216	4.50e-04	1.85 (1.31-2.62)	N Shore	Body;TSS200	C20orf134;NECAB3
cg00478435	32254706	3.02e-05	1.92 (1.41-2.61)	N Shore	1stExon;5'UTR;Body	C20orf134;NECAB3
cg07470512	32255052	3.99e-04	1.76 (1.29-2.40)	Island	1stExon;5'UTR;Body	C20orf134;NECAB3
cg03904042	32255491	6.64e-04	1.75 (1.27-2.42)	Island	1stExon;Body	C20orf134;NECAB3
cg14921437	32255988	8.12e-05	1.95 (1.40-2.71)	Island	1stExon;Body	C20orf134;NECAB3
cg13403462	32256071	8.36e-05	1.98 (1.41-2.78)	S Shore	1stExon;3'UTR;Body	C20orf134;NECAB3

OS, Model B (luminal A), chr18:11688987 – 11690145^1^ **(corresponding to** Figure 3D **)**						
Probe	Position	*P*-value	HR(95% CI)	Island Context	Gene Region	Gene
cg06070749	11688988	1.45e-03	1.72 (1.23-2.41)	N Shore	TSS200	GNAL
cg19488391	11689024	7.38e-05	1.89 (1.38-2.59)	N Shore	TSS200	GNAL
cg02931159	11689032	1.64e-05	1.90 (1.42-2.54)	N Shore	TSS200	GNAL
cg12253819	11689062	1.92e-05	1.96 (1.44-2.67)	Island	TSS200	GNAL
cg15616946	11689206	3.51e-06	1.92 (1.46-2.53)	Island	1stExon;5'UTR	GNAL
cg15653282	11689218	1.00e-06	2.15 (1.58-2.93)	Island	1stExon;5'UTR	GNAL
cg09331011	11689284	1.35e-04	1.83 (1.34-2.50)	Island	1stExon;5'UTR	GNAL
cg12585806	11689613	2.31e-06	2.08 (1.54-2.83)	Island	1stExon	GNAL
cg22318872	11690145	1.70e-02	1.47 (1.07-2.03)	S Shore	Body	GNAL

Subtype- or menopausal-status-specific genomic regions associated with survival outcomes were identified using the summary statistics (*P*-values) results from the site-specific analyses of TCGA breast cancer DNAm data. The top four differentially methylated regions containing at least four CpG loci were selected based on adjusted combined *P*-values of a region (See Materials and Methods). All of these regions were identified to be specific to luminal A subtype since the summary statistics results were from the site-specific analyses using Model B. Model B tested the association between beta-values and the survival outcomes under the assumption that the association could be modified by tumor subtype; the model was adjusted for age, race, stage, menopausal status, tumor purity and cell type proportion. ^1^A region containing CpG loci is coded based on the locations of starting and ending CpG loci in terms of their chromosome number and base-pair positions. chr, chromosome; HR, hazards ratio; UTR, untranslated region; TSS200, 0-200 base-pairs upstream of transcription start site; TSS1500, 200-1500 base-pairs upstream of transcription start site; N shore, northern shore.

### Validation results

**FIGURE 4:**
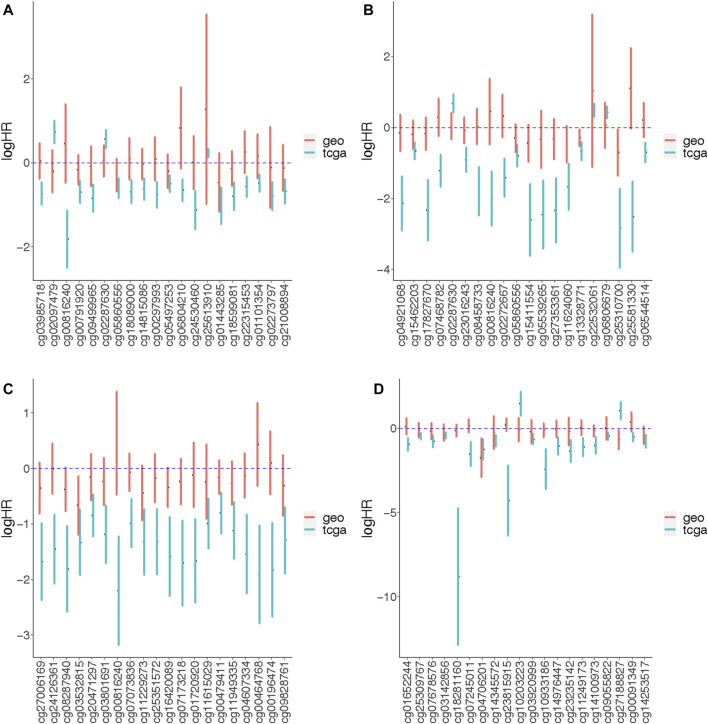
The effect measures of association between DNAm and overall survival outcomes quantified from genome-wide association analyses of TCGA and GEO data. The CpG probes labeled on the *x*-axis are top 20 significant probes from the TCGA site-specific analyses, which are arranged from left to right in an increasing order by *p*-values. We compare effect measures of association between DNAm and overall survival quantified from site-specific analyses of TCGA and GEO data using **(A)** Model A, **(B)** Model B (luminal A-specific), **(C)** Model B (luminal B-specific) and **(D)** Model B (basal-like-specific). Model A tested the association of beta-values and the survival outcomes stratified by the tumor subtypes (no effect measure modifications); Model B tested the association between beta-values and the survival outcomes under the assumption that the association could be modified by tumor subtype. The models for TCGA data adjusted for age, race, stage, menopausal status, tumor purity and cell type proportion. The models for GEO data adjusted for age, tumor purity and cell type proportion.

Validation of results of TCGA site-specific analyses was carried out on Illumina 450K array data profiled from BC tissue of 180 patients available in GEO database (GSE72308) (Jeschke et al., 2017). We were only able to analyze associations with OS since GEO dataset did not include any other common type of survival outcome. These validation models did not adjust for race and stage, or evaluate effect modification by menopausal status as these characteristics were missing from the GEO data. Tumor purity and cell proportion of samples were estimated and included in the models. The age of a majority of subjects was less than 60 years old in both TCGA and GEO. At alpha level of 0.05, there were no significant age differences between the two datasets (*p*-value = 0.148) based on binary categorization of the age variable. More than 50 percent of tumor subtypes of TCGA samples was luminal A, whereas the subtypes of GEO samples were relatively balanced across luminal A, luminal B and basal-like subtypes (Table 1). The median follow-up times for all-cause mortality were not significantly different (*p*-value = 0.789) between the two datasets at alpha level of 0.05. We compared the top 20 most significant (arranged by increasing order of *p*-values) associations with OS identified from TCGA site-specific analyses to those estimated among the GEO dataset. While the directions of associations were similar, most did not reach statistical significance, potentially due to the smaller sample size of the GEO dataset. Associations with similar magnitudes between the two datasets include the overall association with DNAm at cg05497253 (Figure 4A), as well as the luminal A subtype-specific associations at cg05860556 and cg13328771 (Figure 4B), and the basal-like-subtype specific associations at cg04706201 and cg14253517 (Figure 4D).

**FIGURE 5:**
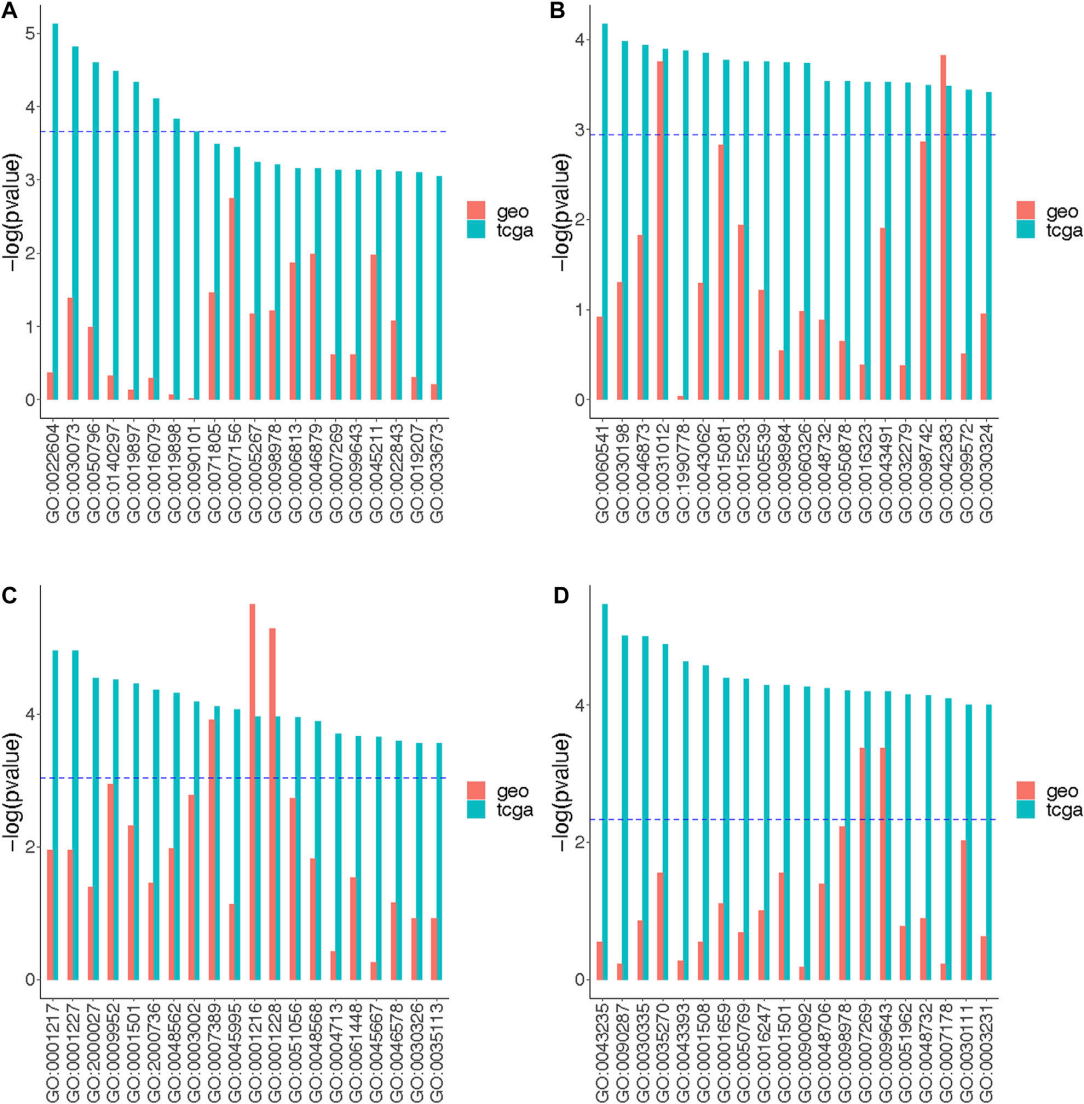
The strengths of the functional pathway enrichment by the association signals between DNAm and overall survival outcomes. The top 20 significantly enriched GO terms representing functional pathways are labeled on the *x*-axis. The GO terms are arranged from left to right in an increasing order by adjusted *p*-values obtained from the gene set enrichment analysis. The enriched pathways were identified using the strengths of the signals indicated by *p*-values obtained from the site-specific analyses of TCGA and GEO data using **(A)** Model A, **(B)** Model B (luminal A-specific), **(C)** Model B (luminal B-specific) and **(D)** Model B (basal-like-specific). Model A tested the association of beta-values and the survival outcomes stratified by the tumor subtypes (no effect measure modifications); Model B tested the association between beta-values and the survival outcomes under the assumption that the association could be modified by tumor subtype. The models for TCGA data adjusted for age, race, stage, menopausal status, tumor purity and cell type proportion. The models for GEO data adjusted for age, tumor purity and cell type proportion. The blue dotted lines indicate the maximum *unadjusted* enrichment *p*-value of GOs whose *adjusted* enrichment *p*-values were at most 0.05.

Further, we compared *p*-values for the 20 most significantly enriched GOs identified from the TCGA site-specific analysis results to the *p*-values of matching GOs identified from the GEO site-specific analysis results (Figure 5). Several GOs seemed to be consistent across datasets. Specifically, for the luminal A subtype-specific associations, GO:0031012 (extracellular matrix) and GO:0042383 (sarcolemma) showed consistent results between TCGA and GEO (Figure 5B). For the luminal B subtype-specific associations, GO:0007389 (pattern specification process), GO:0001216 (DNA-binding transcription activator activity), and GO:0001228 (DNA-binding transcription activator activity) showed consistent results between TCGA and GEO (Figure 5C). For the basal-like subtype-specific associations, GO:0007269 (neurotransmitter secretion) and GO:0099643 (signal release from synapse) showed consistent results across datasets (Figure 5D).

## Discussion

In this study, we detected subtype- or menopausal-status-specific changes in DNAm associated with OS and PFI for BC across the genome. We also investigated functional pathways enriched among the site-specific associations with these BC survival outcomes. Several previous studies that aimed to identify shifts in DNAm associated with BC prognosis acknowledged these relationships may differ across tumor subtypes (Hao et al., 2017; de Almeida et al., 2019; Du et al., 2019; Liu et al., 2020; Tao et al., 2020). Unfortunately, these prior studies did not directly evaluate effect modification by subtype or menopausal status. Xiao et al. examined associations between subtype-specific DNAm and OS (Xiao et al., 2018). However, their analyses were limited to subjects with luminal BC and did not examine effect modification based on menopausal status and associations between DNAm and BC progression. Tumor subtype and menopausal status inform patient treatment, and are associated with different prognoses and treatment responses. In fact, alterations in DNAm can induce resistance to chemotherapy or hormone therapy for BC patients, and recent developments in epigenetic therapies combined with conventional therapies seem promising (Vietri et al., 2021; Schröder et al., 2022). Thus, studying the subtype- and menopausal-status-specific DNAm sites in association with BC prognosis could potentially improve the precision of BC prognoses and help to identify potential drug targets. To the best of our knowledge, our study is the first to report subtype- or menopausal-status-specific genome-wide significant probes that can potentially be utilized as prognostic BC biomarkers. These CpG probes have not been reported in the previous tissue-based BC studies that aimed to find DNAm biomarkers for BC prognosis prediction, indicating potential existence of lurking DNAm sites for BC prognosis that can be detected only after accounting for effect modification by subtype or menopausal status. This was illustrated in our site-specific results where we have found multiple CpG probes whose DNAm levels were significantly associated with BC progression or all-cause mortality, only in models that incorporated effect modification by subtype or menopausal status.

A study by de Almeida et al, (2019) examined genome-wide differential methylation between BC tissue and matched normal tissue using TCGA’s 450K array data. They reported that all of CpG loci associated with poor prognosis (with respect to overall survival) were hypermethylated. In the current study, two CpG loci were associated with increased risk of BC progression with higher methylation levels. In addition, they have found adjusting for ER status of samples rendered some of identified prognostic CpG probes no longer significantly associated with survival outcomes. This indicates an evidence of effect measure modification by subtype on methylation levels on survival outcomes, which consistent with the current study’s findings.

Some of the genes mapped to the significant probes identified in this study have previously been associated with BC initiation and progression. Higher DNAm at cg00175150, a probe within the 3’ untranslated region (UTR) of *ECM1*, was associated with a reduced hazard of PFI among post-menopausal women. The ECM1 protein is secreted by HER2-overexpressing cancer cells, leading to positive downstream effects on tumor migration and invasion, facilitating tumor progression (Steinhaeuser et al., 2020). Among women with the luminal A subtype, higher DNAm at cg15462203, a probe within the gene body of disheveled segment polarity protein 1 (*DVL1*), was associated with a reduced hazard of all-cause mortality. The *DVL1* gene plays a role in activating Wnt transcriptional pathways (Lee et al., 2008; Paclíková et al., 2017), which regulate cellular functions such as cell migration, proliferation and stem cell renewal. *DVL1* has been shown to be overexpressed in primary breast tumors compared to non-cancerous breast tissues, and DVL1 protein has been found to be more present in the cytoplasm of cancer cells compared to that of normal epithelial cells in breast tumors (Nagahata et al., 2003). Also, *DVL1* is involved in regulation of *CYP9A1* transcripts in a promoter specific and cell-type specific manner. Encoded by *CYP9A1*, aromatase enzyme converts androgen into estrogen; hence, aromatase enzyme is considered a main driver of hormone-dependent breast tumors (Castro-Piedras et al., 2018). Knockout of *DVL1* in hormone receptor positive BC cell lines increased the total aromatase transcript levels (Castro-Piedras et al., 2018). Moreover, in the same type of cell lines, the knockout of *DVL1* showed a trend of increased estradiol levels compared to non-target controls, although it was not statistically significant. These findings suggest the tumor suppressive role of *DVL1* by reducing estrogen production *via* regulation of *CYP9A1* for hormone receptor positive BC cells. Higher DNAm at cg03216043, a probe within the gene body of *Dynamin 2* (*DNM2*), was found to decrease risk of BC progression among luminal B tumors. DNM2 plays a role in driving cell migration and invasion in cancer cells (Eppinga et al., 2012; Chernikova et al., 2018). Knockdown of *DNM2* impairs DNA repair mechanisms of tumor cells in mice (Wang et al., 2017). In retrospective analysis, lower expression of *DNM2* was associated with favorable response to chemotherapy for hormonal receptor negative and triple-negative BC patients. In our study, cg06956006 was mapped to the *ACLY* gene, and was related to a lower risk of BC progression given higher levels of methylation. Upregulation of *ACLY* gene, which plays an important role in synthesis of fatty acids in cancer proliferation, is associated with BC and its recurrence (Yancy et al., 2007; Wang et al., 2017; Chen et al., 2020). The hazard of BC progression was greater among patients with higher DNAm at cg17735983, a probe mapping to *MZF1*. *MZF1* is involved in the signaling pathways of HER2+ BC and implicated in development of more aggressive BC (Brix et al., 2020).

We detected several enriched GOs among our site-specific associations with OS and PFI, which differed by tumor subtype and menopause status. Of note, a gene set associated with GO:0050796, tied to insulin secretion, included *KCNS3* gene, which was found in our site-specific analysis and mapped by cg18703983. This particular probe was significantly associated with PFI, suggesting that the hypermethylation of this probe is associated with BC prognosis, in part *via* the regulation of insulin secretion.

Our DMR analysis identified several regional changes in DNAm that were associated with different subtype or menopausal status for both OS and PFI endpoints. The top four significant DMRs intersected with *ELAC1*, *MZF1*, *NECAB3*, and *GNAL*, respectively. Both *ELAC1* and *MZF1* were associated with PFI among the luminal A tumor samples in site-specific analyses. *ELAC1* and *GNAL* have not been reported to be linked with BC. However, *NECAB3* was identified to be tumorigenic by promoting normoxic glycolysis in non-breast cancer cell lines (Nakaoka et al., 2016).

We note several limitations of our study. Our validation analysis was limited to 180 individuals, for whom key characteristics including race, tumor stage and menopausal status were missing. Thus, we were unable to adjust for these characteristics, which may have contributed to the lack of significant associations with OS. Moreover, while BC samples of TCGA subjects were collected before any adjuvant and neoadjuvant therapies, BC samples from the validation dataset were collected after patients have undergone the therapies, possibly leading to perturbed DNAm levels. Non-etheless, we were able to identify probes with consistent associations between two the two datasets based on magnitudes and directions of estimated coefficients. Moreover, we found several cellular functions associated with OS for luminal A, luminal B, and basal-like subtypes across the two studies. Another limitation is that our study included relatively fewer luminal B or basal-like tumors, and a majority of patients was post-menopausal. We might be able detect more genome-wide significant associations specific to luminal B and basal-like subtypes and pre- and peri-menopausal women with larger sample size. Due to these smaller strata, we were not able to simultaneously evaluate effect modification by tumor subtype and menopausal status. Since pre-menopausal patients have different BC prognoses compared to post-menopausal patients (Goldhirsch et al., 2011; Keegan et al., 2012; Lian et al., 2017), stratified analysis by both tumor subtype and menopause status could improve the precision of BC prognosis. We also did not have information on patient treatment status or types of treatments. These can be an important factor affecting survival outcomes of patients. Lastly, different clinical sites where BC patients were treated and recruited could potentially affect the survival outcomes, but this information was not available to us.

Despite those limitations, our study had several strengths. These include adjustment for estimated tumor purity and cell proportions of each sample, which may confound the associations. In addition, we fitted models stratified by subtypes and menopause status, yielding probes associated with two survival outcomes, OS and PFI, in subtype- or menopausal-status-specific manner.

Overall, our study found that specific patterns of DNAm were associated with BC prognosis, namely, OS and PFI, and that these associations differed by molecular subtype or menopausal status. We were also able to identify genomic regions and functional pathways that were specific to molecular subtype or menopausal status. These findings warrant additional replication studies in larger, independent datasets as well as further investigations of the functional implications of these patterns of DNAm. Our detected genome-wide-associated-CpG loci could improve prognosis prediction for BC patients and contribute to more tailored therapeutic regimens.

## Data Availability

Publicly available datasets were analyzed in this study. This data can be found here: TCGA DNA methylation data (https://portal.gdc.cancer.gov), TCGA survival data for breast cancer (https://gdc.cancer.gov/about-data/publications/PanCan-Clinical-2018), DNA methylation data for validation analysis (https://www.ncbi.nlm.nih.gov/geo/query/acc.cgi?accandequals; GSE72308), Cell-type-specific methylation matrix for TCGA breast cancer (https://genboree.org/theCommons/projects/edec/documents).
