# Supplementary material for: DNA methylation patterns associated with breast cancer prognosis that are specific to tumor subtype and menopausal status
Source: Front Genet. 2023 Mar 2;14:1133443. doi: 10.3389/fgene.2023.1133443 (PMC10018014; doi:10.3389/fgene.2023.1133443)
Supplement: Supplementary file 1 [file DataSheet1.docx]

Table S1: Selected differentially methylated region

| PFI, Model B (luminal A), chr18:48494200 – 48494958 **(corresponding to Figure S4A)** | | | | | | |
| --- | --- | --- | --- | --- | --- | --- |
| Probe | Position | *P*-value | HR(95% CI) | Island Context | Gene Region | Gene |
| cg16121470 | 48494201 | 1.72e-06 | 1.82 (1.42-2.32) | N Shore | TSS200 | ELAC1 |
| cg10678486 | 48494218 | 5.47e-08 | 1.84 (1.48-2.30) | N Shore | TSS200 | ELAC1 |
| cg22532061 | 48494536 | 6.67e-07 | 1.56 (1.31-1.86) | Island | 5'UTR | ELAC1 |
| cg21461196 | 48494958 | 6.80e-07 | 1.89 (1.47-2.42) | S Shore | 5'UTR | ELAC1 |
| PFI, Model B (luminal A), chr19:59073901 – 59074507 **(corresponding to Figure S4B)** | | | | | | |
| Probe | Position | *P*-value | HR(95% CI) | Island Context | Gene Region | Gene |
| cg26482665 | 59073902 | 5.55e-05 | 1.99 (1.42-2.78) | Island | Body | LOC100131691;MZF1 |
| cg05014211 | 59074005 | 1.99e-05 | 2.12 (1.50-2.99) | Island | Body | LOC100131691;MZF1 |
| cg19363466 | 59074265 | 4.15e-04 | 1.89 (1.33-2.69) | Island | Body | LOC100131691;MZF1 |
| cg08447733 | 59074308 | 1.96e-05 | 2.15 (1.51-3.05) | Island | Body | LOC100131691;MZF1 |
| cg17735983 | 59074482 | 3.01e-08 | 2.44 (1.78-3.35) | Island | Body | LOC100131691;MZF1 |
| cg00577109 | 59074507 | 2.55e-05 | 1.99 (1.44-2.74) | Island | Body | LOC100131691;MZF1 |
| PFI, Model B (luminal A), chr20:32254215 – 32256071 **(corresponding to Figure S4C)** | | | | | | |
| Probe | Position | *P*-value | HR(95% CI) | Island Context | Gene Region | Gene |
| cg12710480 | 32254216 | 4.50e-04 | 1.85 (1.31-2.62) | N Shore | Body;TSS200 | C20orf134;NECAB3 |
| cg00478435 | 32254706 | 3.02e-05 | 1.92 (1.41-2.61) | N Shore | 1stExon;5'UTR;Body | C20orf134;NECAB3 |
| cg07470512 | 32255052 | 3.99e-04 | 1.76 (1.29-2.40) | Island | 1stExon;5'UTR;Body | C20orf134;NECAB3 |
| cg03904042 | 32255491 | 6.64e-04 | 1.75 (1.27-2.42) | Island | 1stExon;Body | C20orf134;NECAB3 |
| cg14921437 | 32255988 | 8.12e-05 | 1.95 (1.40-2.71) | Island | 1stExon;Body | C20orf134;NECAB3 |
| cg13403462 | 32256071 | 8.36e-05 | 1.98 (1.41-2.78) | S Shore | 1stExon;3'UTR;Body | C20orf134;NECAB3 |
| OS, Model B (luminal A), chr18:11688987 – 11690145 **(corresponding to Figure S4D)** | | | | | | |
| Probe | Position | *P*-value | HR(95% CI) | Island Context | Gene Region | Gene |
| cg06070749 | 11688988 | 1.45e-03 | 1.72 (1.23-2.41) | N Shore | TSS200 | GNAL |
| cg19488391 | 11689024 | 7.38e-05 | 1.89 (1.38-2.59) | N Shore | TSS200 | GNAL |
| cg02931159 | 11689032 | 1.64e-05 | 1.90 (1.42-2.54) | N Shore | TSS200 | GNAL |
| cg12253819 | 11689062 | 1.92e-05 | 1.96 (1.44-2.67) | Island | TSS200 | GNAL |
| cg15616946 | 11689206 | 3.51e-06 | 1.92 (1.46-2.53) | Island | 1stExon;5'UTR | GNAL |
| cg15653282 | 11689218 | 1.00e-06 | 2.15 (1.58-2.93) | Island | 1stExon;5'UTR | GNAL |
| cg09331011 | 11689284 | 1.35e-04 | 1.83 (1.34-2.50) | Island | 1stExon;5'UTR | GNAL |
| cg12585806 | 11689613 | 2.31e-06 | 2.08 (1.54-2.83) | Island | 1stExon | GNAL |
| cg22318872 | 11690145 | 1.70e-02 | 1.47 (1.07-2.03) | S Shore | Body | GNAL |
| PFI, Model B (luminal A), chr15:72667882 – 72668568 **(corresponding to Figure S4E)** | | | | | | |
| Probe | Position | *P*-value | HR(95% CI) | Island Context | Gene Region | Gene |
| cg16391783 | 72667883 | 2.17e-05 | 1.65 (1.31-2.08) | N Shore | Body;TSS1500 | C15orf34;HEXA |
| cg12593608 | 72668042 | 3.29e-05 | 1.60 (1.28-1.99) | Island | Body;TSS1500 | C15orf34;HEXA |
| cg08331842 | 72668114 | 5.34e-04 | 1.50 (1.19-1.88) | Island | 1stExon;TSS1500 | C15orf34;HEXA |
| cg22062265 | 72668130 | 5.30e-04 | 1.47 (1.18-1.83) | Island | 1stExon;TSS1500 | C15orf34;HEXA |
| cg18093693 | 72668265 | 4.21e-03 | 1.38 (1.11-1.73) | Island | 1stExon;TSS200 | C15orf34;HEXA |
| cg13587740 | 72668269 | 1.91e-04 | 1.54 (1.23-1.92) | Island | 1stExon;TSS200 | C15orf34;HEXA |
| cg20540327 | 72668275 | 2.17e-03 | 1.41 (1.13-1.75) | Island | 1stExon;TSS200 | C15orf34;HEXA |
| cg00334370 | 72668350 | 2.28e-05 | 1.57 (1.27-1.93) | Island | 1stExon;5'UTR;TSS200 | C15orf34;HEXA |
| cg05805038 | 72668413 | 1.20e-04 | 1.53 (1.23-1.89) | Island | 1stExon;5'UTR;TSS200 | C15orf34;HEXA |
| cg02760766 | 72668543 | 1.41e-05 | 1.62 (1.31-2.02) | Island | Body;TSS200 | C15orf34;HEXA |
| cg10943348 | 72668568 | 5.02e-04 | 1.47 (1.18-1.82) | Island | Body;TSS200 | C15orf34;HEXA |
| OS, Model A (luminal A), chr11:119455229 – 119455808 **(corresponding to Figure S4F)** | | | | | | |
| Probe | Position | *P*-value | HR(95% CI) | Island Context | Gene Region | Gene |
| cg14417372 | 119455230 | 8.38e-04 | 1.46 (1.17-1.82) | Island |  |  |
| cg16196984 | 119455341 | 3.03e-05 | 1.75 (1.35-2.28) | Island |  |  |
| cg11431820 | 119455500 | 4.99e-06 | 1.56 (1.29-1.89) | Island |  |  |
| cg20304210 | 119455551 | 4.68e-06 | 1.68 (1.34-2.09) | Island |  |  |
| cg02882264 | 119455645 | 1.99e-03 | 1.22 (1.07-1.38) | Island |  |  |
| cg05526905 | 119455808 | 2.63e-06 | 1.64 (1.34-2.02) | Island |  |  |
| OS, Model B (luminal A), chr2:97526804 – 97527586 **(corresponding to Figure S4G)** | | | | | | |
| Probe | Position | *P*-value | HR(95% CI) | Island Context | Gene Region | Gene |
| cg12522144 | 97526805 | 4.61e-05 | 0.62 (0.49-0.78) | N Shore | Body | SEMA4C |
| cg11060194 | 97526918 | 3.23e-04 | 0.63 (0.49-0.81) | N Shore | Body | SEMA4C |
| cg10517096 | 97527074 | 1.35e-04 | 0.69 (0.57-0.83) | N Shore | Body | SEMA4C |
| cg05192533 | 97527490 | 2.02e-05 | 0.62 (0.49-0.77) | Island | Body | SEMA4C |
| cg17343483 | 97527523 | 8.03e-05 | 0.68 (0.56-0.82) | Island | Body | SEMA4C |
| cg17818613 | 97527586 | 3.70e-04 | 0.70 (0.58-0.85) | Island | Body | SEMA4C |
| PFI, Model A, chr15:72667882 – 72668543 **(corresponding to Figure S4H)** | | | | | | |
| Probe | Position | *P*-value | HR(95% CI) | Island Context | Gene Region | Gene |
| cg16391783 | 72667883 | 1.24e-04 | 1.38 (1.17-1.64) | N Shore | Body;TSS1500 | C15orf34;HEXA |
| cg12593608 | 72668042 | 1.47e-04 | 1.36 (1.16-1.59) | Island | Body;TSS1500 | C15orf34;HEXA |
| cg08331842 | 72668114 | 3.79e-04 | 1.36 (1.15-1.61) | Island | 1stExon;TSS1500 | C15orf34;HEXA |
| cg22062265 | 72668130 | 3.35e-04 | 1.36 (1.15-1.60) | Island | 1stExon;TSS1500 | C15orf34;HEXA |
| cg18093693 | 72668265 | 4.70e-04 | 1.35 (1.14-1.61) | Island | 1stExon;TSS200 | C15orf34;HEXA |
| cg13587740 | 72668269 | 1.58e-04 | 1.39 (1.17-1.64) | Island | 1stExon;TSS200 | C15orf34;HEXA |
| cg20540327 | 72668275 | 1.79e-04 | 1.38 (1.17-1.63) | Island | 1stExon;TSS200 | C15orf34;HEXA |
| cg00334370 | 72668350 | 2.25e-05 | 1.42 (1.21-1.66) | Island | 1stExon;5'UTR;TSS200 | C15orf34;HEXA |
| cg05805038 | 72668413 | 8.82e-05 | 1.38 (1.18-1.63) | Island | 1stExon;5'UTR;TSS200 | C15orf34;HEXA |
| cg02760766 | 72668543 | 6.53e-05 | 1.39 (1.18-1.63) | Island | Body;TSS200 | C15orf34;HEXA |
| PFI, Model B (luminal B), chr19:10928171 – 10928696 **(corresponding to Figure S4I)** | | | | | | |
| Probe | Position | *P*-value | HR(95% CI) | Island Context | Gene Region | Gene |
| cg27648270 | 10928172 | 8.69e-06 | 0.21 (0.11-0.42) | OpenSea | Body;TSS200 | DNM2;MIR199A1 |
| cg18544365 | 10928178 | 4.11e-05 | 0.31 (0.18-0.54) | OpenSea | Body;TSS200 | DNM2;MIR199A1 |
| cg23047544 | 10928211 | 2.99e-05 | 0.21 (0.10-0.44) | OpenSea | Body;TSS200 | DNM2;MIR199A1 |
| cg02660440 | 10928233 | 4.61e-04 | 0.40 (0.24-0.67) | OpenSea | Body;TSS200 | DNM2;MIR199A1 |
| cg06754197 | 10928322 | 7.19e-04 | 0.32 (0.16-0.62) | OpenSea | Body;TSS200 | DNM2;MIR199A1 |
| cg02907064 | 10928327 | 6.84e-05 | 0.27 (0.14-0.51) | OpenSea | Body;TSS200 | DNM2;MIR199A1 |
| cg23068797 | 10928549 | 6.97e-06 | 0.26 (0.14-0.47) | OpenSea | Body;TSS1500 | DNM2;MIR199A1 |
| cg03216043 | 10928639 | 3.87e-08 | 0.15 (0.08-0.29) | OpenSea | Body;TSS1500 | DNM2;MIR199A1 |
| cg13965612 | 10928696 | 2.64e-03 | 0.57 (0.39-0.82) | OpenSea | Body;TSS1500 | DNM2;MIR199A1 |
| OS, Model B (luminal A), chr7:129007901 – 129008179 **(corresponding to Figure S4J)** | | | | | | |
| Probe | Position | *P*-value | HR(95% CI) | Island Context | Gene Region | Gene |
| cg22584802 | 129007902 | 3.42e-05 | 0.09 (0.03-0.29) | OpenSea | Body;TSS200 | AHCYL2 |
| cg08738403 | 129007916 | 2.06e-04 | 0.15 (0.06-0.41) | OpenSea | Body;TSS200 | AHCYL2 |
| cg17827670 | 129008130 | 7.65e-08 | 0.10 (0.04-0.23) | OpenSea | 1stExon;5'UTR;Body | AHCYL2 |
| cg11961845 | 129008179 | 9.02e-06 | 0.08 (0.03-0.25) | OpenSea | 1stExon;5'UTR;Body | AHCYL2 |

chr, chromosome; HR, hazards ratio; UTR, untranslated region; TSS200, 0-200 bp upstream of transcription start site; TSS1500, 200-1500 bp upstream of transcription start site; N shore, northern shore; Models correspond to models used in site-specific analysis from which *P*-values of probes were obtained

Figure S1: **(A)** overall survival Model A; **(B)** overall survival Model B (luminal

A); **(C)** progression-free interval Model A; **(D)** progression-free interval Model B (luminal A);

**(E)** progression-free interval Model B (luminal B); **(F)** progression-free interval Model C (post-

menopause)

| **A**  **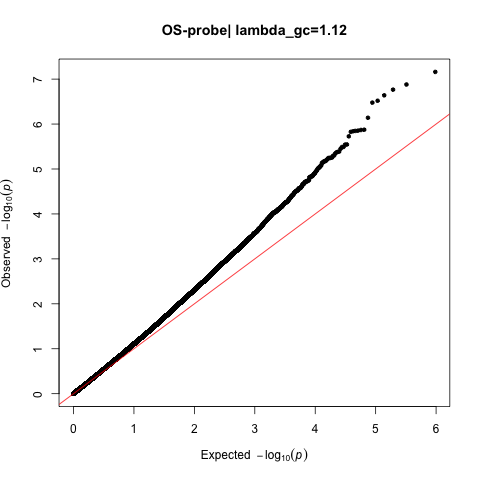** | **B**  **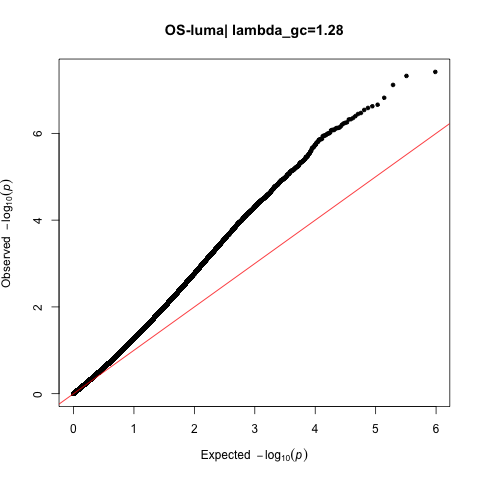** |
| --- | --- |
| **C**  **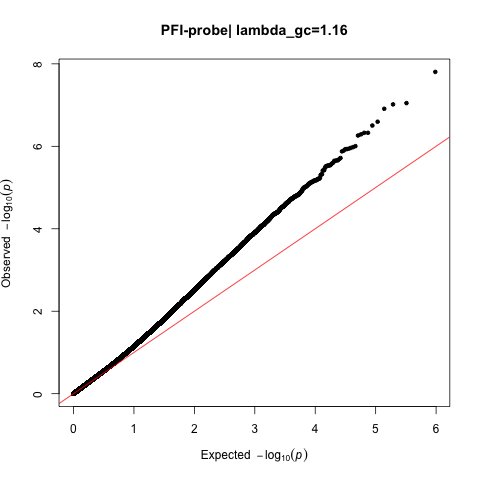** | **D**  **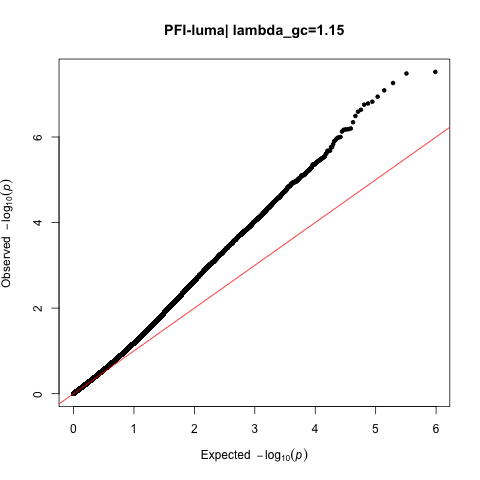** |
| **E**  **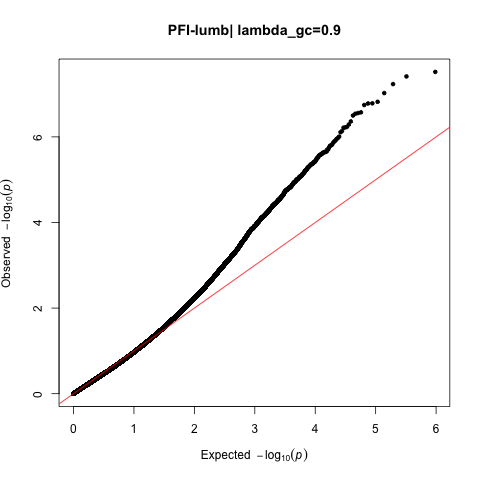** | **F**  **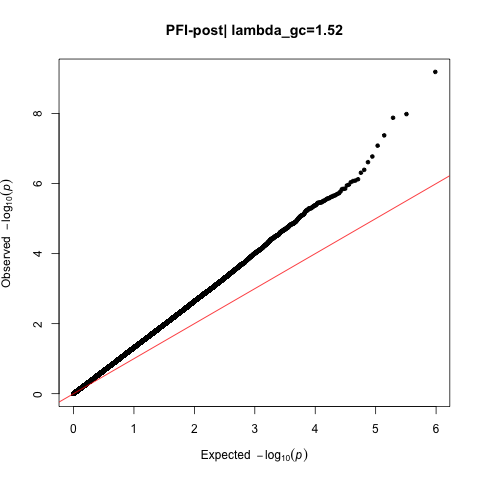** |

Figure S2: Functional pathway analysis results of overall survival endpoint.

Each subplot shows top 10 results of functional pathway analysis by adjusted *P*-value for

each model. **(A)** Model A; **(B)** Model B (luminal A); **(C)** Model B (luminal B); **(D)** Model B

(basal); **(E)** Model C (post-menopause); **(F)** Model C (pre- and peri-menopause)

| **A**  **** | **B**  **** |
| --- | --- |
| **C**  **** | **D**  **** |
| **E**  **** | **F**  **** |

Figure S3: Functional pathway analysis results for progression-free interval

endpoint. Each subplot shows top 10 results of functional pathway analysis by adjusted

P-value for each model. **(A)** Model A; **(B)** Model B (luminal A); **(C)** Model B (luminal B); **(D)**

Model B (basal); **(E)** Model C (post-menopause); **(F)** Model C (pre- and peri-menopause)

| **A**  **** | **B**  **** |
| --- | --- |
| **C**  **** | **D**  **** |
| **E**  **** | **F**  **** |

Figure S4. Selected differentially methylated region. Each subplot describes a region in the genome around identified DMR (flanking 2000 bp). These subplots are top ten DMRs (top four already appears in the main manuscript) based on adjusted *P*-values (See Methods) with at least four probes within a DMR. **(A)** PFI, Model B (luminal A), chr18:48494200 – 48494958; **(B)** PFI, Model B (luminal A), chr19:59073901 – 59074507; **(C)** PFI, Model B (luminal A), chr20:32254215 – 32256071; **(D)** OS, Model B (luminal A), chr18:11688987 – 11690145; **(E)** PFI, Model B (luminal A), chr15:72667882 – 72668568; **(F)** OS, Model A (luminal A), chr11:119455229 – 119455808; **(G)** OS, Model B (luminal A), chr2:97526804 – 97527586; **(H)** PFI, Model A, chr15:72667882 – 72668543; **(I)** PFI, Model B (luminal B), chr19:10928171 – 10928696; **(J)** OS, Model B (luminal A), chr7:129007901 – 129008179. The first track shows CpG island context, the second track shows chromatin state, the third track shows gene context (Ensembl), and the fourth track shows P-values of the probes for each subtype. A red box represents a significant DMR. A vertical bar on the chromosome schematic locates plotted region. Chromatin state color scheme: electric lime, transcribed 3’ preferential and enhancer (Enh), or transcribed 5’ preferential and Enh, or transcribed and weak Enh; red, active transcription start site (TSS); orange red, promoter (Prom) upstream/downstream TSS; yellow, weak Enh or primary H3K27ac possible Enh; white, quiescent; orange, active enhancer; pink, poised Prom; dark purple, bivalent Prom; light green, weak transcription; green, transcribed or strong transcription; gray, repressed polycomb.

| **A**  **** | **B**  **** |
| --- | --- |
| **C ** | **D**  **** |
| **E**  **** | **F**  **** |
| **G**  **** | **H**  **** |
| **I**  **** | **J**  **** |

Figure S5: Comparison of genome-wide significant probe association *P*-values and estimated effect measures across different models. probe, Model A; luma, Model B (luminal A); lumb, Model B (luminal B); basal, Model B (basal-like); preperi, Model C (pre- and peri-menopausal); post, Model C (post-menopausal).

| **A**  **** | **B**  **** |
| --- | --- |
| **C**  **** | **D** |
| **E**  **** | **F** |
| **G**  **** | **H** |
| **I**  **** | **J** |
| **K**  **** | **L** |
| **M** | **N** |
| **O**  **** | **P** |
| **Q**  **** | **R** |
| **S**  **** | **T** |
| **U**  **** | **V** |
| **W** | **X** |
| **Y**  **** | **Z** |
| **I**  **** | **II** |
| **III** | **IV** |
| **V**  **** | **VI** |
| **VII** | **VIII** |
